# Effect of COVID-19 lock down on teenage pregnancies in Northern Uganda: an interrupted time series analysis

**DOI:** 10.1186/s12978-023-01707-8

**Published:** 2023-11-04

**Authors:** Jimmy Patrick Alunyo, David Mukunya, Agnes Napyo, Joseph K. B. Matovu, David Okia, Wanume Benon, Francis Okello, Ally Hassan Tuwa, Daniel Wenani, Ambrose Okibure, Godfrey Omara, Peter Olupot-Olupot

**Affiliations:** 1 Department of Community and Public Health, Busitema University, Mbale, Uganda; 2 School of Medicine, Kabale University, Kabale, Uganda; 3 Mable Clinical Research Institute, Mbale, Uganda

**Keywords:** Interrupted time series analysis, Teenage pregnancy, Adolescent

## Abstract

**Background:**

Pregnancy and childbirth complications are the leading cause of death among girls aged 15–19 years globally, with low- and middle-income countries (LMICs) accounting for 99% of global maternal deaths of women aged 15–49 years. Despite teenage pregnancies declining in many developing countries in recent years, the COVID-19 period intensified the problem and altered the trend for most countries. We determined the effect of the COVID-19 lockdown on the teenage pregnancy trend in Pakwach district, Uganda, to understand its magnitude in our study population.

**Methods:**

Using interrupted time series analysis (ITS), sometimes known as quasi-experimental time series analysis. We constructed a time series of the first ANC service utilization records for girls aged 10–19 years in Pakwach district, Uganda, and conducted an interrupted series analysis. We compared the two periods of March 2019 to March 2020 and March 2020 to March 2021. We used Stata 15 to conduct our analysis, performed OLS, and plotted the results.

**Results:**

The teenage pregnancy trend before the lockdown was decreasing by − 0.203 pregnancies per month, but in the first month after the institution of the lockdown (March 20, 2020), there was an increase in the teenage pregnancy rate of 13.9 pregnancies [95% CI: − 33.6 to 61.5], which corresponds to an increase in the monthly trend in teenage pregnancies (relative to the period before the COVID-19 lockdown trend) of 1.53 girls per month.

**Conclusion:**

Teenage pregnancies increased during the lockdown. This slight increase depicted the impact of the pandemic on the teenage pregnancy trend associated with the COVID-19 outbreak. The government needs to focus on intervention to reduce this trend and avoid any further increases.

## Background

Pregnancy and childbirth complications are the leading cause of death among girls aged 15–19 years globally, with LMICs accounting for 99% of global maternal deaths of women aged 15–49 years [1]. Although the rates of adolescent pregnancy have decreased in recent years around the world [2], the emergency of the COVID-19 pandemic and its effects, like lockdown, have increased the rates of teenage pregnancy. The prolonged school closure brought by the lockdown had a significant impact on the past trends in teenage pregnancies in many countries in Africa. Lockdown-related school closures frequently have a significant detrimental effect on the sexual and reproductive health and rights of adolescent girls [3, 4]. Nevertheless, when it comes to preventing teenage pregnancies, the most successful intervention initiatives have consistently gone beyond solely addressing reproductive health. These programs encompass broader aspects such as educational opportunities and vocational skills, primarily offered within school settings. During the COVID-19 period, the inactive school environment led to a significant increase in teenage pregnancies. Future research is needed to understand the risk factors for teenage pregnancy during the lockdown and their prevalence to inform interventions. In Uganda, prior to the pandemic, the prevalence of teenage pregnancies had remained stagnant since 2006, as reported in the UDHS and by UNPA [5]. The highest recorded rate was observed in 2000/2001 at 31%. However, in the subsequent years, there was a gradual decline of 6% to reach 25% in 2006, followed by a further 1% decrease in 2011 [6]. Nevertheless, the rate saw a slight increase of 1% in 2016, making it the highest in East Africa. Despite the overall stagnation with the outbreak of the COVID-19 pandemic, experts worry about the potential change in trend from stagnation to an increasing trend due to the effect of the lockdown [7]. The impact of the lockdown and its effects, like prolonged school closure, on teenage pregnancy needs to be evaluated. School closures during the 2014 Ebola outbreak in west African countries increased teenage pregnancy trends [4]. In Uganda, there is a paucity of data to understand the effects of the COVID-19 lockdown on teenage pregnancy. Therefore, in this study, we used an interrupted time series analysis and estimated the effects of the lockdown on teenage pregnancy during the COVID-19 pandemic period in Pakwach District, northern Uganda. We compared the trend in teenage pregnancy before and during COVID-19 in Pakwach District, Northern Uganda.

## Methods

### Study design

This study utilized an interrupted time series design used in epidemiology, public health, and other fields to assess the impact of an intervention or policy change over time. For our study, the researchers collected data at multiple time points, both before and during the COVID-19 lockdown. This design allowed us to examine the trends in the outcome of teenage pregnancy over time and evaluate whether the lockdown had a significant and sustained effect on the teenage pregnancy trend.

### Study period

The researcher considered one year before the institution of the lockdown and one year after the institution of the lockdown. In total, the researcher collected data for 25 months, with March 2020 as the starting point when our intervention was implemented.

### Study setting

The study area was Pakwach District, located in the West Nile Region of northern Uganda. According to Uganda Bureau of Statistics figures, the district had a total population of 158,037 in 2014 and was projected to increase to 181,400 by 2018, and 51.7% of these are females. Antenatal care first-visit attendance is very high in our study setting, above 95%, as reported in the DHIS2. The District Health Information System 2, which collects aggregate data on ANC attendance for women, was then accessed to retrieve our dataset.

### Target population

The target population in this study was a record of teenage girls aged 10–19 who got pregnant and attended their first ANC visits to health facilities in Pakwach district from March 2019 to March 2021.

### Data collection

In our analysis, the researcher obtained teenage pregnancy data directly from the District Health Information System 2 (DHIS2) database, which is accessible at the DHO office. We specifically searched and retrieved data related to the first antenatal care (ANC) service for adolescent girls aged 10–19 during the period from March 2019 to March 2021. The indicators required for this analysis (period name, ANC first visit for women less than 15 years, and ANC first visit for women aged 15–19 years) were correctly specified by the researcher to the district biostatistician at the DHO office. Using the district Health Information System 2 dataset, which is one of the most reliable databases for keeping aggregate health records in Uganda, the researcher logged into the system and retrieved data as per our specified indicators into an Excel sheet. The extracted records the researcher collected over a span of 25 months, encompassing both pre-lockdown and lockdown periods. During that period, the district recorded a total of 4422 pregnancies among adolescents during these two selected periods.

### Testing for model fit

Before conducting our data analysis using the interrupted time series (ITS) design, the researcher performed several important tests to assess key characteristics. These tests include visual examination by plotting the time series data, evaluating descriptive statistics, testing model assumptions to validate the chosen ITSA model, and checking for autocorrelation, which assesses the correlation and proximity of collected data. The researcher also conducted tests for secular trends, examining whether the dataset exhibited consistent increases or decreases over time regardless of any intervention, as well as for seasonality. Therefore, in our analysis, the researcher diligently examined all these essential attributes in our dataset and generated the necessary graphs, particularly autocorrelation graphs. Furthermore, the researcher examined the overdispersion in our data to ensure it adhered to the assumption of the Poisson distribution, where the variance is equal to the mean, and confirmed that our data were suitable for the chosen model. The autocorrelation checks ultimately guided our decision to employ an OLS model, specifically the Newey model with a specified lag of 1.

### Statistical analysis

The researcher conducted two-level analyses to compare teenage pregnancy trends before and during COVID-19 in Pakwach. In the first analysis, the researcher loaded the data, declared the dataset as a panel in Stata, and specified a single group ITS analysis. For this analysis, the intervention start was set for March 20th, 2020, when the COVID-19 lockdown was instituted in Uganda. The researcher then conducted an ITS analysis using OLS and plotted the results. See Table 1.

**Table 1:** Provides the output of Interrupted Time Series Analysis results

Preg	Coefficient	Newey-west std.err	t	P >|t|	[95% conf. interval]
_t	− 0.2027972	1.559033	− 0.13	0.898	− 3.444983 3.039389
_x722	13.94272	22.87324	0.61	0.549	− 33.62479 61.51023
_x_t722	1.532468	2.738131	0.56	0.582	− 4.161787 7.226722
_cons	155.2821	9.697319	16.01	0.000	135.1154 175.4487

Firstly, to compare the trend in teenage pregnancy before and during COVID-19, the researcher utilized the Stata itsa (interrupted time series analysis) command. After importing the data to Stata, the researcher started the analysis by ensuring that our time variable was in the proper format, e.g., 722 became 2020 m3 (March). After that, the researcher then declared the new time variable created to become a time set.

**Fig. 1:**
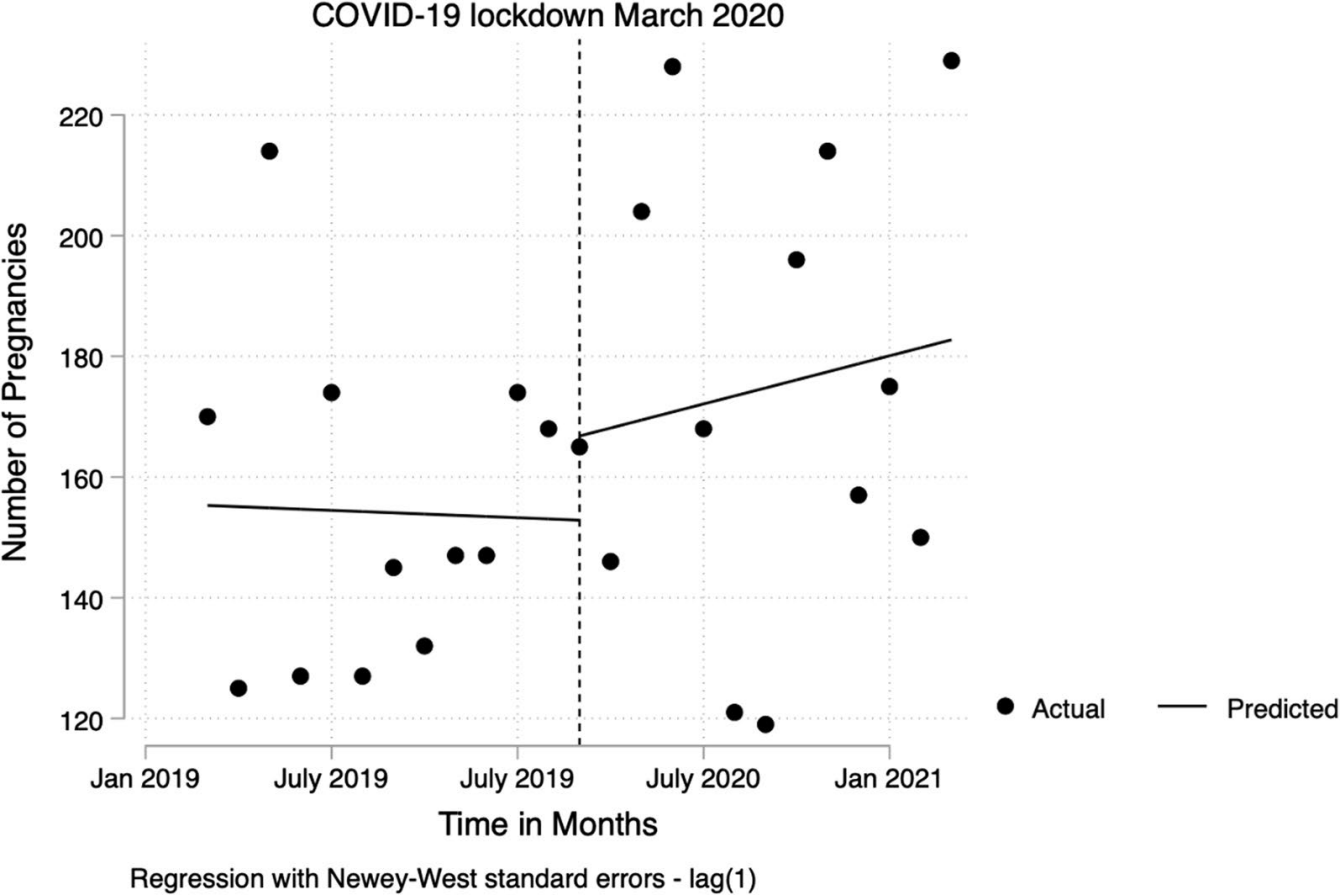
Showing results of OLS plots

At this stage, the researcher performed some descriptive analysis to just check our trends by summarizing our variable Preg. This was followed by a time-series analysis conducted in Stata using the itsa command. The result was presented in a table and a graph. (See Table 1 and Fig. 1). More importantly, we made sure that our model fit by checking for autocorrelation, using p < 0.05 at any lag to show autocorrelation, finding no autocorrelation in the data, indicating that there was no need to adjust for it, and plotting its graphs.

In the second analysis, the researcher used the poison regression command to estimate the effect of the COVID-19 lockdown on teenage pregnancy trends. In this poison regression analysis, our outcome became the number of pregnancies, intervention (COVID-19 lockdowns), and time (_n of time periods chronologically arranged). After specifying all conditions of the family of Poisson regression and plotting a log (link) eform for it, the researcher generated the predicted values based on this model and produced a plot of the model along with a scatter graph. The researcher then generated the counterfactual by removing the effect of the intervention (_b [smokban]) for the post-intervention period and adding the counterfactual to our plot.

To ensure the soundness of the model, the researcher further checked for autocorrelation by examining the autocorrelation and partial autocorrelation functions. Additionally, the researcher also adjusted for seasonality as one of the important attributes in the analysis. The results were presented in the form of a table and graphs (See Table 2 and Fig. 1).

**Table 2:** Presents results for generalized linear poison output

Preg	IRR	OIM std.err	z	p >|z|	[95% conf. interval]
Covid_status	1.078935	0.1725095	0.48	0.635	0.7886744–1.4760022
Time	1.003963	0.0110966	0.36	0.720	0.9824581–1.025949
_cons	150.2396	14.08758	53.45	0.000	125.0171–180.5508

## Results

As shown in Table 2 below, the starting level of teenage pregnancy rate was estimated at 155 pregnancies per month, and the rate appears to significantly decrease every month prior to March 2020 by − 0.203 pregnancies. [95% CI: − 4.335 to 4.218]. pregnancy, in the first month after the institution of the COVID-19 lockdown (March 20th, 2020), there appeared to be an increase in the teenage pregnancy rate of 13.9 pregnancies [95% CI: − 33.6 to 61.5], which corresponds to an increase in the monthly trend (relative to the period before the COVID-19 lockdown trend) of 1.53 girls per month [95% CI: − 4.2 to 7.2]. After the institution of COVID-19 lockdowns, the monthly trend of teenage pregnancies in Pakwach district (relative to the period before the COVID-19 lockdown trend) increased by 1.53 pregnancies per month [95% CI: − 3.4 to 6.0].

In Fig. 1, from the result of the ordinary logistic regression plot, we see that the lockdown period is associated with an increase in the number of teenage pregnancies. Before the lockdown, the trend was decreasing. After the institution of the lockdown, the trend started to increase relative to the declining trend we saw before the lockdown. As shown in Table 2 below, the starting level of teenage pregnancy rate was estimated at 155 pregnancies per month, and the rate appears to significantly decrease every month prior to March 2020 by − 0.203 pregnancies [95% CI: − 4.335 to 4.218]. However, in the first month after the institution of the COVID-19 lockdown (March 20th, 2020), there appeared to be an increase in the teenage pregnancy rate of 13.9 pregnancies [95% CI: − 33.6 to 61.5], which corresponds to an increase in the monthly trend (relative to the period before the COVID-19 lockdown trend) of 1.53 girls per month [95% CI: − 4.2 to 7.2]. After the institution of COVID-19 lockdowns, the monthly trend of teenage pregnancies in Pakwach district (relative to the period before the COVID-19 lockdown trend) increased by 1.53 pregnancies per month [95% CI: − 3.4 to 6.0].

In Fig. 1, from the result of the ordinary logistic regression plot, we see that the lockdown period is associated with an increase in the number of teenage pregnancies. Before the lockdown, the trend was decreasing. After the institution of the lockdown, the trend started to increase relative to the declining trend we saw before the lockdown.

### Analysis two: Poisson

**Fig. 2:**
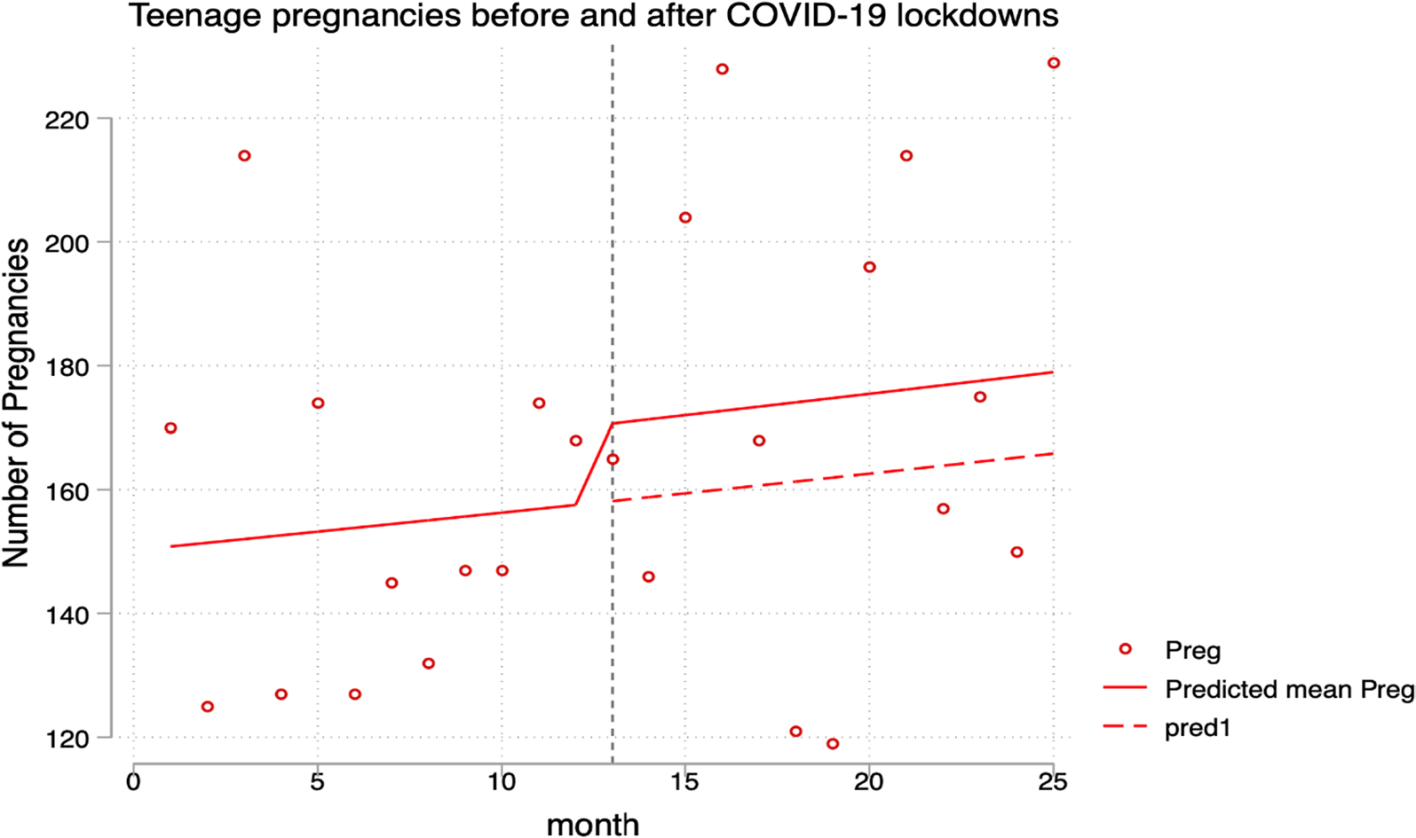
Showing results generalized poison regression plot

To estimate the effect of the COVID-19 lockdowns on teenage pregnancies, we fitted a generalized linear model of the Poisson family with a log link and robust variance estimation. After running the model, we found out that the COVID-19 lockdown period resulted in an 8% increase in teenage pregnancies, as shown in Table 2 and Fig. 2 below. However, this estimate was a bit imprecise in terms of statistical significance.

## Discussion

Generally, in Pakwach District, teenage pregnancy rates are higher (22.4%), as reflected in the Uganda Demographic Health Survey 2016 [8], Although this is the aggregate statistic for all the West Nile districts and not only for Pakwach district, it is still very high. However, during the COVID-19 period, we witnessed an increasing trend, as captured in our data.

The starting level of the teenage pregnancy trend in the district was estimated at 155 pregnancies per month. It was significantly decreasing every month before the lockdown. However, after the institution of the lockdown, this trend started increasing in the first month after the lockdown and continued monthly at a rate of 1.53 girls relative to the period before COVID-19. The increase we saw could have been because of the lockdown environment that exposed adolescent girls to many risk factors associated with pregnancy while at home. These findings agree with that of another report published by Plan International during the 2014 Ebola outbreak in West Africa where lockdown was instituted as a public health measure [9]. Additionally, it also agrees with Zulaika and others in Kenya, who also found school-age girls suffering from the effect of covenant-19 containment to have 2 times the risk of becoming pregnant [10].

This new trend corresponds to an 8% increase in teenage pregnancies in the district, and we think that factors like prolonged school closure, sexual abuse, limited access to contraceptives and others imposed by the lockdown environment on teenage girls could explain this increase. Our finding in this analysis is similar to studies conducted in West Africa during the Ebola outbreak in 2014, where a lockdown instituted to prevent Ebola led to increased teenage pregnancy rates and a shift in the trend of teenage pregnancy in school-going adolescents due to prolonged school closures [9].

Data elsewhere in Uganda also indicate that the teenage pregnancy rate in girls aged 10–19 years during the COVID-19 lockdown plummeted [11]. Even though factors that contributed to the high increase are yet to be investigated, in the population of Pakwach, the lockdown contributed an 8% increase, which we attributed to the lockdown environments and other factors.

### Strength and limitations of our study

This ITS analysis was the first of its kind to estimate the effect of the pandemic lockdown on teenage pregnancies in the district and this result can be taken as a true picture of the teenage pregnancy situation in the district. However, there is a chance that we might have missed some numbers for girls who didn’t access the health facility during their pregnancy, but based on statistics of the first ANC attendance for the district which stands very high above 95%, the researcher thinks our proxy first ANC attendance dataset used in this analysis gave us a sufficient data points required to determine and arrived at the estimate we found in this analysis.

## Conclusion

In conclusion, almost no studies have utilized interrupted time series analysis to compare teenage pregnancy trends and rates in Uganda before, and this study is the first of its kind that we think can be used as a benchmark for many intervention studies in this area of teenage pregnancy. But all in all, we found this design very valuable in evaluating teenage pregnancy interventions, trends, and rates, and therefore we do recommend it for future studies in this area.

## Data Availability

The data collected for this research is available in case it is needed.

## Abbreviations

ITSA: Interrupted Time Series Analysis
DHIS2: District Health information system
WHO: World Health Organization
COVID-19: Corona Virus Disease 2019
